# Aberrant expression of KDM1A inhibits ferroptosis of lung cancer cells through up-regulating c-Myc

**DOI:** 10.1038/s41598-022-23699-4

**Published:** 2022-11-10

**Authors:** Can Lu, Yuan Cai, Wei Liu, Bi Peng, Qiuju Liang, Yuanliang Yan, Desheng Liang, Zhijie Xu

**Affiliations:** 1 Department of Pathology, Xiangya Hospital, Central South University, Changsha, China; 2 Center for Medical Genetics & Hunan Key Laboratory of Medical Genetics, School of Life Sciences, Central South University, Changsha, China; 3 Department of Orthopedic Surgery, The Second Hospital University of South China, Hengyang, China; 4 Department of Pharmacy, Xiangya Hospital, Central South University, Changsha, China; 5 National Clinical Research Center for Geriatric Disorders, Xiangya Hospital, Central South University, Changsha, China

**Keywords:** Cancer, Cell death

## Abstract

Ferroptosis is a cell death process caused by metabolic dysfunction with the feature of aberrant iron accumulation. Emerging studies have identified that ferroptosis is an important biological function involving in the tumorigenesis, and targeting ferroptosis could provide promising therapeutic targets for lung cancer. However, such therapeutic strategies show limited therapeutic effect owing to drug resistance and other unknown underlying mechanisms. In this study, lysine-specific demethylase 1 (LSD1/KDM1A) was found to be significantly upregulated in lung cancer cells and tissues. The patients with KDM1A downregulation displayed the good prognosis. Using gene set enrichment analysis (GSEA), we demonstrated that KDM1A-associated genes might participate in the regulation of cell ferroptosis and Myc signaling in lung cancer. Knockdown of KDM1A inhibited the level of c-Myc and increased the concentration of malondialdehyde (MDA) and irons in human lung cancer cells H1299 and A549. Downregulation of c-Myc could facilitate KDM1A knockdown-mediated ferroptosis. Our study has elucidated the effect of KDM1A/c-Myc regulatory axis in the ferroptosis resistance of lung cancer cells.

## Introduction

Lung cancer has been proved to be the most common cause for cancer-associated death^1,2^. Nowadays, therapy resistance still occurs and leads to the recurrence of lung cancer patients^3^. Recent studies showed that the cisplatin-resistant cancer cells exhibit less sensitive to ferroptosis inducers^4^. Moreover, the clinical use of ferroptosis induction can improve the therapeutic efficacy in cancer patients^5,6^. However, the underlying mechanisms and biological functions of ferroptosis in lung cancer pathogenesis and therapeutic response remain unclear.

Ferroptosis, a novel type of regulatory cell death, triggered by depletion of glutathione and lipid peroxidation^7^. Emerging evidences have suggested that dysregulation of ferroptosis is closely related to tumorigenesis and treatment^8,9^, highlighting the regulation of ferroptosis as a promising anticancer therapeutic strategy^10^. Previous studies have suggested the potential roles of oncogenic c-Myc in ferroptosis^11^. In lung cancer cells, high levels of c-Myc significantly inhibits ferroptosis through directly activating lymphoid-specific helicase^12^. Clarifying the c-Myc signaling networks and the associated factors underlying ferroptosis would be critical to effectively sensitize cancer cells to the ferroptosis‐based therapeutic strategies.

Emerging studies have shown that epigenetic modifications could regulate the expression of signaling molecules involved in the cell death. As an important epigenetic enzyme, lysine specific demethylase 1 (LSD1/KDM1A) plays a key functional role in mediating cancer cell death^13^. Aberrant KDM1A signaling participates in a range of biological processes, including cell proliferation, epithelial-to-mesenchymal transition (EMT) and malignant transformation^14^. Emerging reports have demonstrated that KDM1A is commonly dysregulated in a variety of cancers, suggesting KDM1A as a probable biomarker for cancer development and treatment^15^. However, the underlying mechanisms of KDM1A on the regulation of cell ferroptosis is relatively limited and remains to be explained.

Here, we evaluated the potential roles of KDM1A-c-Myc axis in lung cancer. Based on several public bioinformatic databases, we found that KDM1A was over-expressed in lung cancer tissues. And the patients with upregulated KDM1A displayed unfavorable prognosis. Silencing of KDM1A significantly impeded the cell growth and increased the sensitivity to erastin-induced ferroptosis in A549 and H1299 cells. We also found that blocking KDM1A-c-Myc axis could significantly activate cell ferroptosis. Our data suggested that KDM1A confers resistance to ferroptosis in lung cancer cells through upregulating c-Myc signaling.

## Materials and methods

### Data collection

UALCAN (http://ualcan.path.uab.edu/) and TNMplot (https://tnmplot.com/analysis/), two comprehensive and user-friendly databases, were used to analyze the mRNA and protein levels of KDM1A in cancers^16,17^. Two survival databases, PrognoScan^18^ and Kaplan–Meier plotter^19^, were used to analyze the effect of KDM1A expression in patients’ prognosis, including relapse free survival (RFS), overall survival (OS) and first progression (FP). The expression levels of KDM1A in lung cancer tissues were further analyzed using two datasets from Gene Expression Omnibus (GEO), GSE13213 and GSE31210. The database, cBioPortal, was used to analyzed the co-expressed genes of KDM1A in a lung adenocarcinoma dataset (TCGA, PanCancer Atlas). And the GSEA pathway analysis was performed using Xiantao Xueshu (https://www.xiantao.love/products).

### Cell culture and reagents

Human lung cancer cell lines H1299, A549, H157 and H358 were obtained from the Cancer Research Institute, Central South University, China. Cells were cultured in 37 °C incubator with 5% CO_2_. The cell culture medium for A549 was RPMI-1640 (Procell, Cat#PM150110P) with 10% FBS (Gbico, Cat#10099141C). And the cell culture mediums for H1299, H157 and H358 were Dulbecco’s modified Eagle’s medium (DMEM) (Procell, Cat#PM150210P) with 10% FBS. H1299-shKDM1A and A549-shKDM1A cells were cultured with respective medium supplemented with 1 μg/ml puromycin (Beyotime, Cat#ST551). The exposing concentrations of erastin (Sigma-Aldrich, Cat#E7781) and ferrostatin-1 (APExBIO, Cat#A4371) in lung cancer cells were 5 μM and 10 μM, respectively.

### Lentivirus production and infection

293 T cells were seeded in 6 mm plates and co-transfected with lentivirus package plasmid Pax2 and VSVG the next day. After 48 h incubation at 37 °C, we harvested the virus-containing supernatants. For cell infection, the mixture of virus liquid and polybrene (5 μg/ml) was added into cell culture medium. The sequences for KDM1A shRNAs (shKDM1As) were obtained as previously described^20^ and inserted into the pLKO.1 lentiviral vectors. shKDM1A: GATCCCCAGGAAGGCTCT TCTAGCAATATTCAAGAGATATTGCTAGAAGAGCCTTCCTTTTTTC, TCGAGA AAAAAGGAAGGCTCTTCTAGCAATATCTCTTGAATATTGCTAGAAGAGCCTTCCTGGG; shKDM1A-2: GATCCCCGGAGCTCCTGATTTGACAAAGTTCAAGA GACTTTGTCAAATCAGGAGCTCCTTTTTC, TCGAGAAAAAGGAGCTCCTGA TTTGACAAAGTCTCTTGAACTTTGTCAAATCAGGAGCTCCGGG; shCtrl:GATCCCCAATTGCCACAACAGGGTCGTGTTCAAGAGA, CACGACCCTGCCGTGGCAATTTTTTTC, TCGAGAAAAAAATTGCCA, CAACAGGGTCGTGTCTCTTGAACACGACCCTGCCGTGGCAATTGGG.

### Transient transfection

We purchased c-Myc siRNA from Genepharma (China) according to the previous report^21^. We conducted the transfection of siRNA into cancer cells according to the manufacturer’s introductions of lipofectamine 3000 (Invitrogen, United States). In brief, 1 × 10^6^ cells were seeded in 60 mm plates and performed transfection the next day. After 48 h incubation, the cells were further collected for subsequent experiments.

### Isolation of RNA and real-time polymerase chain reaction

After extraction using Trizol (Invitrogen, Cat#15596018), the total RNA was reverse-transcribed with the reverse transcription kit, PrimeScript 1st strand cDNA synthesis kit (Takara, Cat#6210A). Next, we used real-time polymerase chain reaction (RT-PCR) to analyze the transcriptional levels of KDM1A, c-Myc and HMOX1. The forward and reverse primer sequences are as follows: β-actin: 5′-CATGTACGTTGCTATCCAGGC-3′ and 5′-CTCCTTAATGTCACGCACGAT-3′; KDM1A: 5′-TGACCGGATGACTTCTCAAGA-3′ and 5′-GTTGGAGAGTAGCCTC AAATGTC-3′; c-Myc: 5′-GGCTCCTGGCAAAAGGTCA-3′ and 5′-CTGCGTAGTT GTGCTGATGT-3′. HMOX1: 5′-AAGACTGCGTTCCTGCTCAAC-3′ and 5′-AAAGCCCTACAGCAACTGTCG-3′. We used the 2^−∆∆CT^ method to calculate their relative expression levels.

### Western blot

After extraction using lysis buffer (Thermo Scientific, United States), the total protein were resolved on the SDS-PAGE and transferred onto a nitrocellulose membrane (Millipore, United States). Next, the nitrocellulose membrane was blocked in 5% skimmed milk, and incubated with the indicated primary antibodies at 4 °C overnight. The following antibodies were used: anti-KDM1A (Abcam, United States, Cat#ab17721), anti-c-Myc (Santa Cruz, United States,Cat# sc-40), anti-HMOX1 (Proteintech, United States, Cat# 10701-1-AP) and anti-β-actin (Santa Cruz, United States, Cat# sc-69879). The protein levels were determined using the chemiluminescence reagent (Millipore, USA). According to the molecular weight, the nitrocellulose membrane was cut prior to hybridization with antibodies and visualized using the ChemiDoc XRS system (Bio-Rad, Berkeley).

### Cell viability assay

We performed the cell proliferation assay according to the manufacturer’s introductions of MTS assay kit (B34304, Bimake, United States). In brief, 1 × 10^3^ cells were seeded in 96-well plate. After incubated with erastin (5 μM) orRSL3 (1 μM),ferrostatin-1 (10 μM) for 72 h and MTS solution for 1 h, the optical density of cells was detected at 450 nm with a spectrometer (PerkinElmer, United States).

### Iron levels assay

We detected the concentration of cellular irons according to the manufacturer’s introductions of iron assay kit (Abcam, United States). After treated with ferroptosis inducer erastin (5 μM) or RSL3 (1 μM) for 24 h, cells were rapidly mixed with iron assay buffer. We then removed the insoluble material, and added the iron assay buffer and iron probe into the reaction mixture. At last, the spectrometric absorbance was detected at the wavelength of 593 nm.

### MDA level assay

We detected the concentration of cellular malondialdehyde (MDA) according to the manufacturer’s introductions of lipid peroxidation assay kit (Sigma-Alorich, United States). After treated with ferroptosis inducer erastin (5 μM) or RSL3 (1 μM) for 24 h, cells were lysed on ice by ultrasonication. We then removed the insoluble material, and added thiobarbituric acid (TBA) solution into the samples. At last, the spectrometric absorbance was detected at the wavelength of 532 nm.

### Statistical analysis

All data represented in graphs were shown as mean ± standard deviation (SD). Student’s t-test and one way analysis of variance (ANOVA) were used for the difference comparisons or multivariate analysis. The difference has a statistic meaning (*p < 0.05, **p < 0.01 and ***p < 0.001).

## Results

### KDM1A expression was upregulated in lung cancer

**Figure 1:**
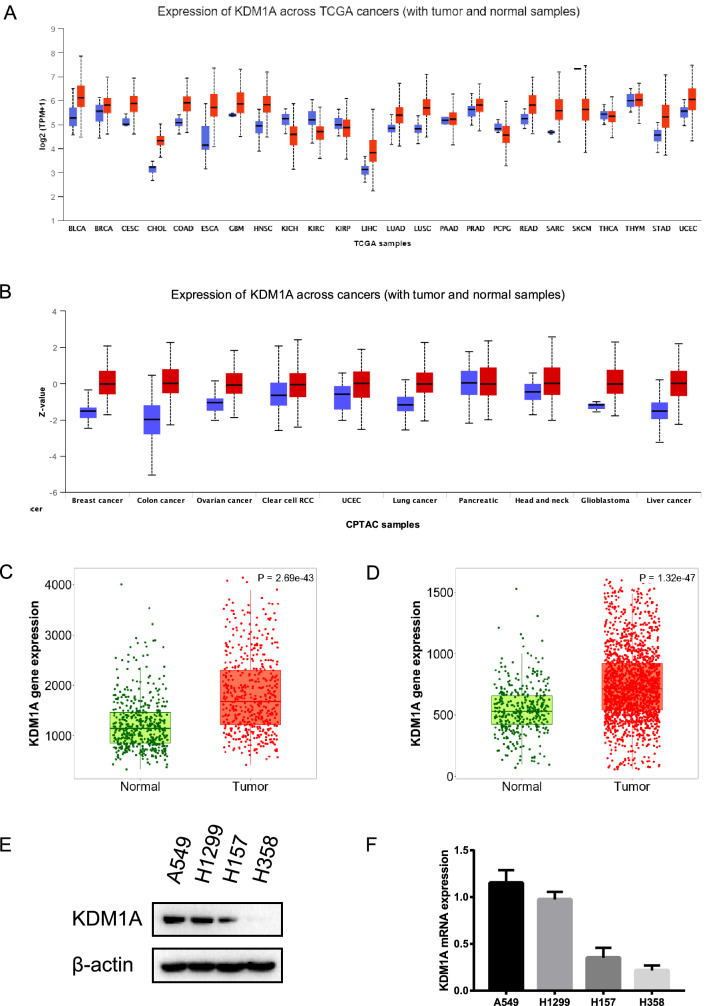
KDM1A was over-expressed in lung cancer tissues and cells. (**A**) Pan-cancer analysis of KDM1A expression profiles using UALCAN database. Red box refers to the tumor and blue box refers to the normal tissues. (**B**) Pan-cancer analysis of KDM1A protein expression in the CPTAC database from UALCAN. Red box refers to the tumor and blue box refers to the normal tissues. (C-D) KDM1A expression was higher than normal tissues analyzed from RNA-Seq data (**C**) and Gene-chip data (**D**) from TNMplot database. (**E**, **F**) The KDM1A protein and mRNA levels in four lung cancer cells, A549, H1299, H157 and H358 cells. According to the molecular weight, the nitrocellulose membrane was cut prior to hybridization with antibodies and the original blots are presented in Supplementary Fig. 6.

Pan-cancer analysis from UALCAN database revealed the upregulated expression levels of KDM1A in majority of cancers, including lung cancer (Fig. 1A,B, Table S1). We also utilized the TNMplot database to demonstrated that KDM1A was overexpressed in lung cancer tissues from RNA-Seq data (Fig. 1C) and Gene-chip data (Fig. 1D). In addition, we detected the expression profiles of KDM1A in several lung cancer cells. We found the significantly over-expressed KDM1A in A549 and H1299 cells (Fig. 1E,F). Thus, A549 and H1299 cells were used for subsequent experiments.

### KDM1A exhibited poor prognosis in patients with lung cancer

**Figure 2:**
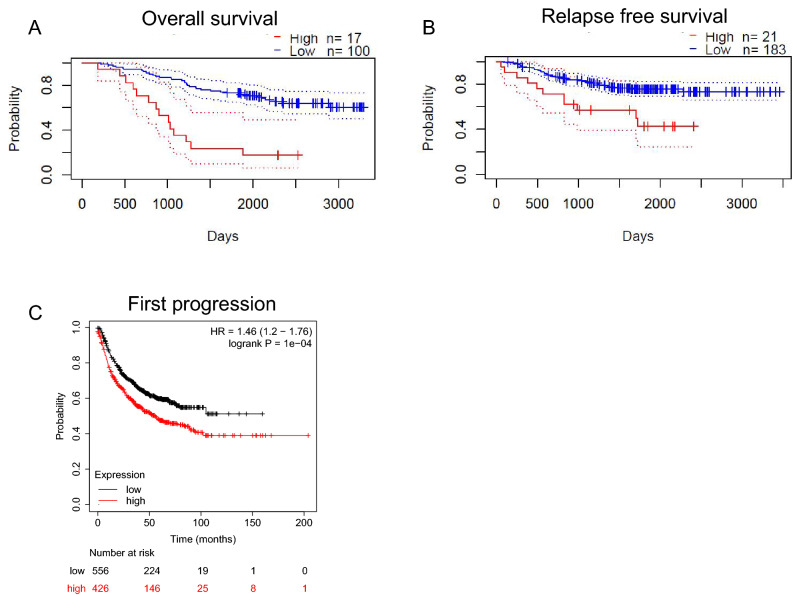
Prognostic values of KDM1A in lung cancer patients. (**A**, **B**) PrognoScan indicated the roles of KDM1A on OS (**A**) and RFS (**B**) in patients with lung cancer. (**C**) Kaplan–Meier plotter indicated the roles of KDM1A on FP in patients with lung cancer.

We then used two survival databases, PrognoScan and Kaplan–Meier plotter, to analyze the effect of KDM1A levels on patients’ prognosis. Of note, the patients with high levels of KDM1A displayed poor OS (Fig. 2A) and RFS (Fig. 2B). Moreover, the patients with low levels of KDM1A displayed slightly favorable FP (Fig. 2C). These findings above-mentioned suggested that the expression of KDM1A was associated with the poor clinical outcomes in patients with lung cancer.

### Knockdown of KDM1A promoted the ferroptosis sensitivity of lung cancers

**Figure 3:**
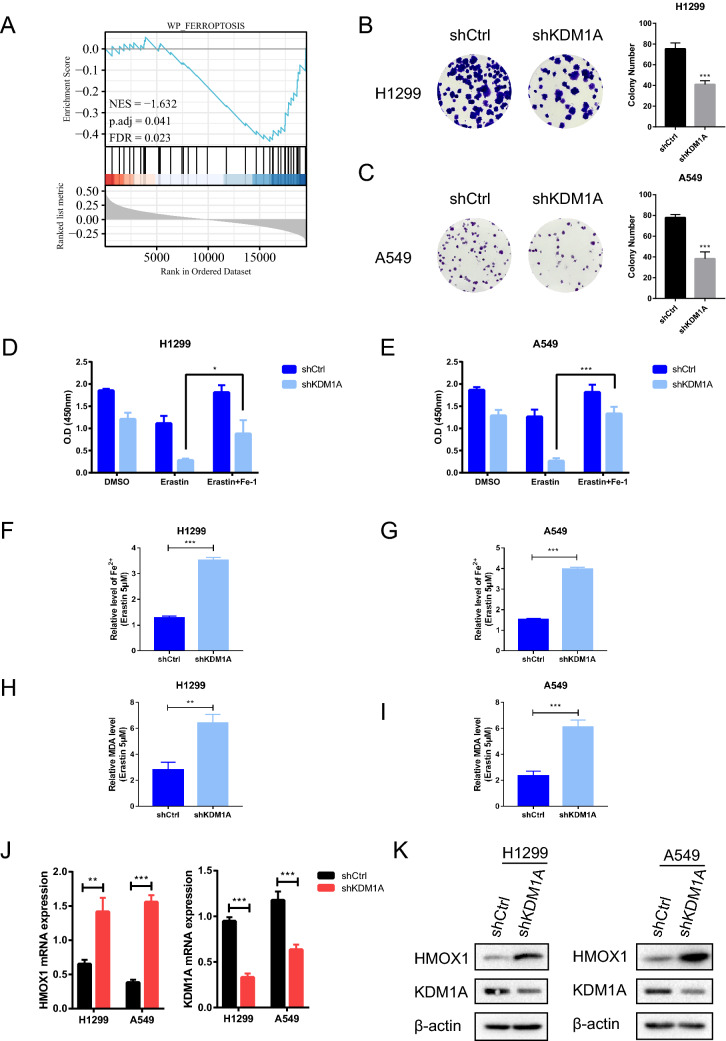
Knockdown of KDM1A could sensitize lung cell to ferroptosis. (**A**) The GSEA pathway analysis revealed that KDM1A co-expressed genes might involve in the cell ferroptosis process. (**B**, **C**) The colony formation assay was performed in H1299 and A549 stable cell lines with KDM1A knockdown. (**D**, **E**) The effect of erastin and ferrostatin-1 on the cell viability of KDM1A knockdown cells. (**F**, **G**) Cellular Fe^2+^ levels were detected in KDM1A knockdown cells treated with erastin. (**H**, **I**) Cellular MDA levels were detected in KDM1A knockdown cells treated with erastin. (**J**, **K**) The HMOX1 expression levels were analyzed in KDM1A knockdown cells. According to the molecular weight, the nitrocellulose membrane was cut prior to hybridization with antibodies and the original blots are presented in Supplementary Fig. 7. The graphs represent mean ± SD, two-tailed, Student’s t-test. N = 3, *p < 0.05; **p < 0.01; **p < 0.001.

The analysis of gene co-expression network is an outstanding approach for identifying the co-expression patterns of candidate genes in different phenotypes, and has been widely used for gene function annotation^22,23^. The co-expressed genes of KDM1A (Table S2) were downloaded from a lung adenocarcinoma dataset, TCGA Pan-Cancer Atlas, to further evaluate the underlying mechanisms regulated by KDM1A co-expression molecules. The GSEA analysis results showed that KDM1A related genes might involve in the cell ferroptosis process (Fig. 3A). Then, we investigated whether KDM1A mediates ferroptosis of lung cancer cells. Firstly, we established H1299 and A549 stable cells with KDM1A gene knockdown. As shown in colony formation assays (Fig. 3B,C), cell growth was significantly decreased in H1299 and A549 cells with KDM1A knockdown. To further understand the roles of KDM1A in cell ferroptosis, we treated the stable KDM1A knockdown A549 and H1299 cell lines with erastin and RSL3, two ferroptosis inducers, and ferrostatin-1 (Fer-1), a ferroptosis inhibitor^24^. The cell viability assays showed that silencing of KDM1A significantly enhanced the lethality of erastin and RSL3 in H1299 and A549 cells. However, Fer-1 treatment could significantly weaken the lethality induced by erastin or RSL3 (Fig. 3D,E, Fig. S3G,H).

Iron accumulation and lipid peroxidation have been proved to be the two primary biochemical characteristics of ferroptosis process. And MDA is one of the end products from lipid peroxidation^9^. Accordingly, erastin treatment increased the concentration of intracellular Fe^2+^ and MDA levels in KDM1A knockdown H1299 and A549 cells (Fig. 3F–I). Inversely, we obtained the downregulated trend of intracellular Fe^2+^ and MDA levels in H1299 KDM1A overexpression cells induced by erastin or RSL3 (Fig. S3C–F). Furthermore, after KDM1A knockdown, RT-PCR was used to analyze the expression of several ferroptosis markers. As shown in Fig. S1 and Fig. 3J, we found that ferroptosis-associated molecule, HMOX1^25^, was significantly up-regulated in KDM1A knockdown lung cancer cells H1299 and A549. Western blot also demonstrated inhibition of KDM1A could increase the HMOX1 protein levels (Fig. 3K). In addition, GEPIA2 database showed that KDM1A expression was negatively correlated with HMOX1 expression in lung cancer (Fig. S2A). These data suggested that inhibition of KDM1A could improve the ferroptosis sensitivity of lung cancer cells.

### KDM1A upregulates c-Myc signaling

**Figure 4:**
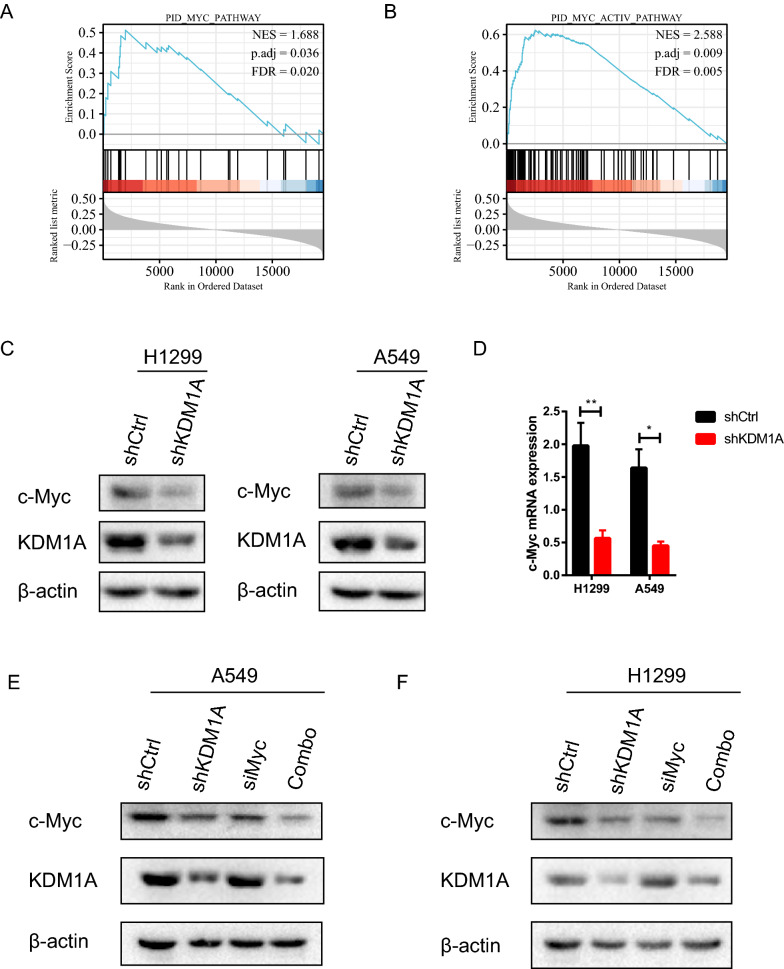
KDM1A knockdown repressed c-Myc expression. (**A**, **B**) The GSEA pathway analysis revealed that KDM1A co-expressed genes might regulate the Myc signaling. (**C**, **D**) The c-Myc protein and mRNA levels were explored in H1299 and A549 stable cell lines with KDM1A knockdown. According to the molecular weight, the nitrocellulose membrane was cut prior to hybridization with antibodies and the original blots are presented in Supplementary Fig. 8. (**E**, **F**) The combination treatment of KDM1A knockdown and c-Myc knockdown synergistically inhibited c-Myc expression in H1299 and A549 cells. According to the molecular weight, the nitrocellulose membrane was cut prior to hybridization with antibodies and the original blots are presented in Supplementary Fig. 8.

The GSEA pathway analysis indicated that KDM1A related genes may participate in Myc activation pathway and Myc pathway (Fig. 4A,B). Then, we investigated whether KDM1A mediates Myc signaling in lung cancer. GEPIA2 database showed that KDM1A expression was positively correlated with Myc expression in lung cancer (Fig. S2B). Depletion of endogenous KDM1A repressed the c-Myc expression levels in H1299 and A549 cells (Fig. 4C,D). Meanwhile, overexpression of KDM1A reversely improved the c-Myc expression levels in H1299 and A549 cells (Fig. S3A,B). Combined knockdown of KDM1A and c-Myc displayed the synergetic inhibitory effect on c-Myc expression (Fig. 4E,F). Additionally, we confirmed the interaction between KDM1A and c-Myc in 293 T cells (Fig. S4). These data collectively suggest that c-Myc might be regulated by KDM1A in lung cancers.

### KDM1A protects lung cancer cells from ferroptosis through c-Myc

**Figure 5:**
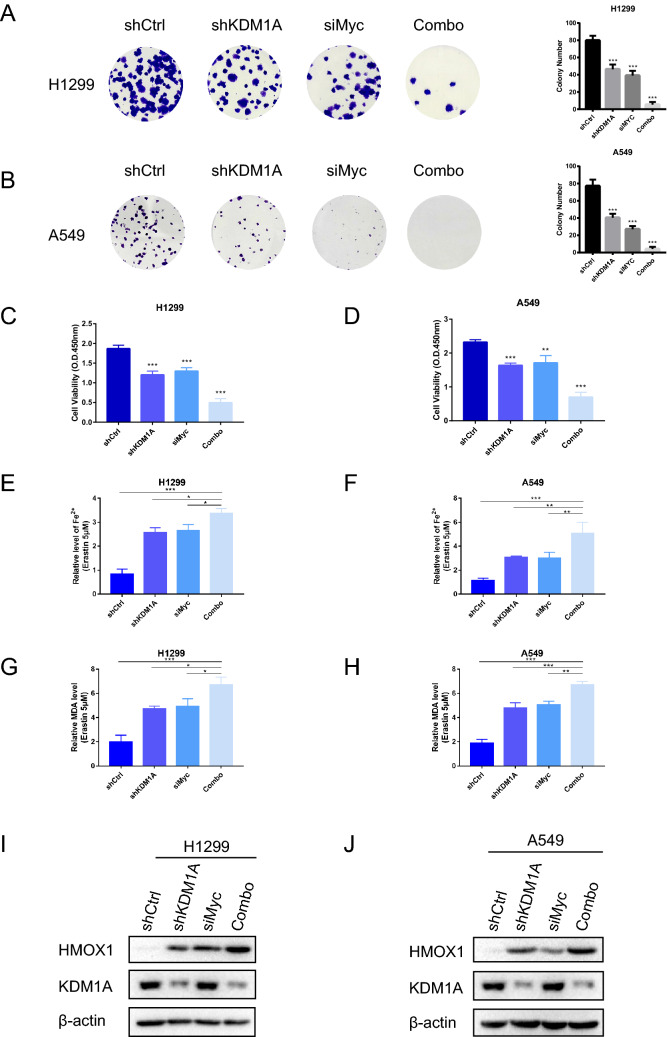
KDM1A-c-MYC axis conferred the resistance to ferroptosis in lung cancer cells. (**A**, **B**) The colony formation assay was performed in lung cancer cells H1299 and A549 with knockdown of KDM1A and c-Myc. (**C**, **D**) The cell viability assay was performed in lung cancer cells H1299 and A549 cells with knockdown of KDM1A and c-Myc. (**E**, **F**) Cellular Fe^2+^ levels were detected in lung cancer cells H1299 and A549 with knockdown of KDM1A and c-Myc treated with erastin. (**G**, **H**) The MDA concentration was detected in lung cancer cells H1299 and A549 with knockdown of KDM1A and c-Myc treated with erastin. (**I**, **J**) The protein levels of HMOX1 were detected in lung cancer cells H1299 and A549 with knockdown of KDM1A and c-Myc. According to the molecular weight, the nitrocellulose membrane was cut prior to hybridization with antibodies and the original blots are presented in Supplementary Fig. 9. The graphs represent mean ± SD, two-tailed, Student’s t-test. N = 3, *p < 0.05; **p < 0.01; **p < 0.001.

Recent studies have displayed the inhibitory effect of c-Myc on ferroptosis^26^. Next, we wanted to elucidate the underlying roles of the KDM1A-c-Myc axis in ferroptosis of lung cancer cells. Combined knockdown of KDM1A and c-Myc synergistically reduced the cell proliferation rates in H1299 and A549 cells (Fig. 5A–D). Moreover, the intracellular Fe^2+^ and MDA levels were significantly increased upon KDM1A and c-Myc knockdown (Fig. 5E–H). Simultaneously, ectopic expression of c-Myc in KDM1A-depleted cells (Fig. S5A,B) treated with erastin or RSL3 completely reversed the decrease of cell growth (Figs. S5C–F, S6E,F) and increase of intracellular Fe^2+^ and MDA levels (Fig. S6A–D). Previous reports have identified HMOX1 as a ferroptosis inducer^27^. Accordingly, c-Myc knockdown further increased the expression level of HOMX1 upon KDM1A knockdown both in H1299 and A549 cells (Fig. 5I,J). These data together indicate that KDM1A protected lung cancer cells against ferroptosis by maintaining c-Myc level.

## Discussion

Nowadays, the 5-year survival rate of lung cancer patients is still unsatisfactory^28^. Exploring the potential therapeutic and prognostic biomarkers are thus urgently needed. Although ferroptosis cell death has been proposed to provide the new hopes for cancer management, the precise roles of ferroptosis in lung cancer development and progression have not been fully elucidated. Previous studies have confirmed the highly-expressed KDM1A in several cancers^29,30^. Accordingly, our study displayed the upregulated KDM1A in lung cancer H1299 and A549 cells. Knockdown of KDM1A obviously suppressed the cell growth and induced the cell death.

The aberrantly expressed c-Myc, a member of MYC gene family, has been proved to participate in the regulation of multiple biological functions in human cancers^31^. It has been reported that c-Myc could regulate the transcription of targeted genes by binding some histone components^32^. Amente et al. showed that c-Myc could directedly bind and recruit KDM1A to the E-box chromatin, promoting the transcriptional activity of target genes^33^. In our study, GSEA pathway analysis indicated that KDM1A might participate in the activation of Myc signaling. And we demonstrated that knockdown of KDM1A could significantly inhibit c-Myc expression levels in lung cancer cells H1299 and A549.

Ferroptosis, a reactive oxygen species-dependent cell death, is characterized with lipid peroxidation and iron accumulation^34,35^. Regarding iron homeostasis regulation, c-Myc has been reported to suppress the expression of iron regulatory proteins, resulting in the accumulation of intracellular iron pool^36^. Accordingly, we noted that c-Myc knockdown as well as KDM1A knockdown significantly increased the expression levels of ferroptosis marker HMOX1 in H1299 and A549 cells. Moreover, knockdown of KDM1A increased ferroptosis of lung cancer cells through downregulating c-Myc expression. KDM1A knockdown or c-Myc knockdown significantly enhanced the lethality induced by erastin in H1299 and A549 cells. Therefore, our findings collectively demonstrated the important roles of KDM1A-c-Myc signaling in the regulation of cell ferroptosis, and revealed a potential therapeutic strategy against lung cancer.

The histone demethylase lysine-specific demethylase 1 (LSD1/KDM1A) can regulate the gene transcription through demethylating the histone H3 lysine 4 (H3K4)^37^. In previous studies, KDM1A down-regulates the antagonist of the canonical Wnt pathway, APC, by demethylating H3K4me1/2 of APC2 promotor, promoting the progression of thyroid cancer^38^. KDM1A reinforces the immunosuppression in hepatocellular carcinoma through demethylating MEF2D and activating PD-L1^39^. KDM1A exerts anti-cancer effect in bladder cancer through demethylating MMP9^40^. In addition, overexpression of KDM1A could effectively protect cell against preterm death^41^. In our study, we found that KDM1A knockdown significantly repressed the expression of c-Myc, leading to the increased ferroptosis sensitivity of lung cancer cells. Therefore, KDM1A might mechanistically mediate the demethylation of c-Myc in lung cancer cells.

In summary, our work revealed the functional roles of KDM1A-c-Myc axis in the regulation of ferroptosis in lung cancer cells. KDM1A depletion could significantly increase the cellular Fe^2+^ concentration and MDA levels through downregulating c-Myc expression, resulting in cell growth inhibition. Therefore, targeting KDM1A could be a potential ferroptosis-based treatment strategy for lung cancer patients.

## Supplementary Information

Supplementary Legends.

Supplementary Figure S1.

Supplementary Figure S2.

Supplementary Figure S3.

Supplementary Figure S4.

Supplementary Figure S5.

Supplementary Figure S6.

Supplementary Figure 6.

Supplementary Figure 7.

Supplementary Figure 8.

Supplementary Figure 9.

Supplementary Figure 10.

Supplementary Figure 11.

Supplementary Figure 12.

Supplementary Table S1.

Supplementary Table S2.

## Data Availability

The datasets generated and/or analyzed during the current study are available in the public databases, such as cBioPortal (http://www.cbioportal.org/), UALCAN (http://ualcan.path.uab.edu/), PrognoScan (http://dna00.bio.kyutech.ac.jp/PrognoScan/index.html)and TNMplot (https://tnmplot.com/analysis/) databases.
