# Supplementary material for: Aberrant expression of KDM1A inhibits ferroptosis of lung cancer cells through up-regulating c-Myc
Source: Sci Rep. 2022 Nov 10;12:19168. doi: 10.1038/s41598-022-23699-4 (PMC9649633; doi:10.1038/s41598-022-23699-4)
Supplement: Supplementary file 1 — Supplementary Legends. [file 41598_2022_23699_MOESM1_ESM.docx]

**Figure S1. The roles of KDM1A knockdown on the expression of several ferroptosis markers.** The expression of several ferroptosis-related markers was detected by RT-PCR in KDM1A knockdown A549 cell lines.

**Figure S2. The correlation between KDM1A and HMOX1/c-Myc in lung cancer.**

(A) GEPIA2 indicated the negative correlation between KDM1A and ferroptosis-related marker HMOX1.

(B) GEPIA2 indicated the positive correlation between KDM1A and Myc.

**Figure S3. KDM1A overexpression increased c-Myc expression and induced resistance to ferroptosis.**

(A-B) The protein levels of c-Myc were detected in lung cancer cells H1299 and A549 with KDM1A overexpression. According to the molecular weight, the nitrocellulose membrane was cut prior to hybridization with antibodies and the original blots are presented in Supplementary Figure 10.

(C-D) Cellular Fe^2+^ levels were detected in H1299 KDM1A overexpression cells treated with erastin or RSL3.

(E-F) Cellular MDA levels were detected in H1299 KDM1A overexpression cells treated with erastin or RSL3.

(G-H) The effect of RSL3 and ferrostatin-1 on the cell viability of KDM1A overexpression cells.

The graphs represent mean ± SD, two-tailed, Student’s t-test. N=3, *p<0.05; **p<0.01; **p<0.001.

**Figure S4. KDM1A interacted with c-Myc.**

The protein interaction between KDM1A and c-Myc. Cell lysates from 293T cells transiently co-transfected with Flag-KDM1A and HA-vector or HA-c-Myc were subjected to immunoprecipitation overnight with anti-Flag agarose beads after 48 hours incubation, and the eluted protein samples were blotted with the indicated antibodies. According to the molecular weight, the nitrocellulose membrane was cut prior to hybridization with antibodies and the original blots are presented in Supplementary Figure 11.

**Figure S5. Overexpression of c-Myc improved cell growth.**

(A-B) The verification of KDM1A knockdown and c-Myc overexpression by western blot. According to the molecular weight, the nitrocellulose membrane was cut prior to hybridization with antibodies and the original blots are presented in Supplementary Figure 12 .

(C-D) The cell viability assay was performed in H1299 and A549 cells with KDM1A knockdown and c-Myc overexpression treated with erastin.

(E-F) The colony formation assay was performed in H1299 and A549 cells with KDM1A knockdown and c-Myc overexpression.

The graphs represent mean ± SD, two-tailed, Student’s t-test. N=3, *p<0.05; **p<0.01; **p<0.001.

**Figure S6. Overexpression of c-Myc induced cell resistance to ferroptosis.**

(A-B) Cellular Fe^2+^ levels were detected in H1299 KDM1A knockdown cells with or without c-Myc overexpression treated with erastin(A) or RSL3(B).

(C-D) Cellular MDA levels were detected in H1299 KDM1A knockdown cells with or without c-Myc overexpression treated with erastin(C) or RSL3(D).

(E-F) The cell viability assay was performed in H1299 and A549 cells with KDM1A knockdown and c-Myc overexpression treated with RSL3.

The graphs represent mean ± SD, two-tailed, Student’s t-test. N=3, *p<0.05; **p<0.01; **p<0.001.

**Table S1. Expression of KDM1A across TCGA and CPTAC cancers.**

Pan-cancer analysis from UALCAN database revealed the upregulated expression levels of KDM1A in majority of cancers, including lung cancer.

**Table S2. The co-expressed genes of KDM1A.**

The co-expressed gene list of KDM1A downloaded from cBioportal database.
