# Supplementary figures and images for: Aberrant expression of KDM1A inhibits ferroptosis of lung cancer cells through up-regulating c-Myc
Source: Sci Rep. 2022 Nov 10;12:19168. doi: 10.1038/s41598-022-23699-4 (PMC9649633; doi:10.1038/s41598-022-23699-4)

Figure S1

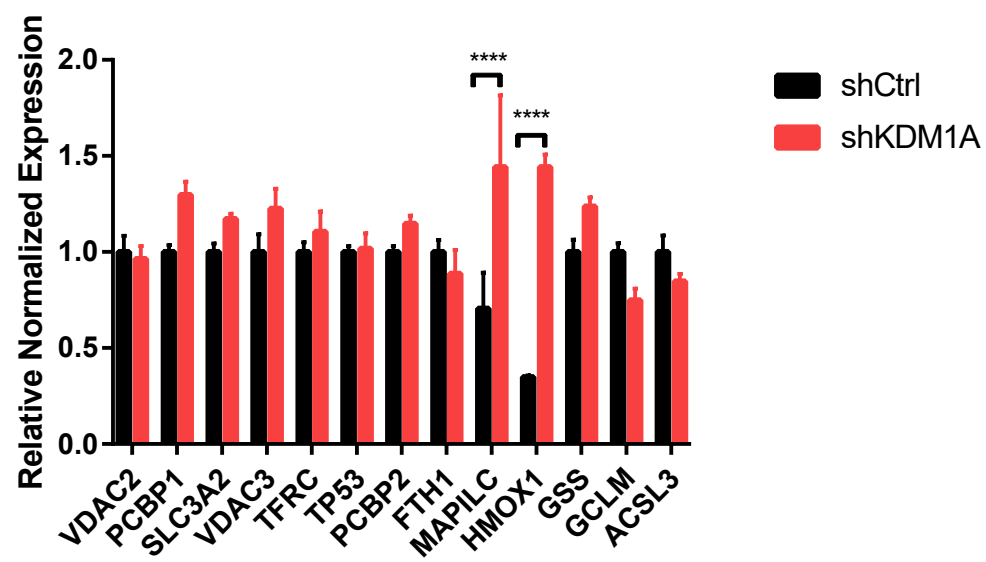

Supplement: Supplementary file 2 — Supplementary Figure S1. [file 41598_2022_23699_MOESM2_ESM.pdf]

Figure S2

A

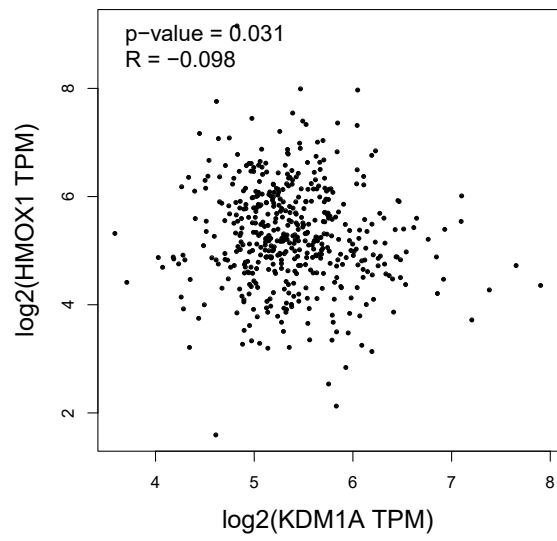

B

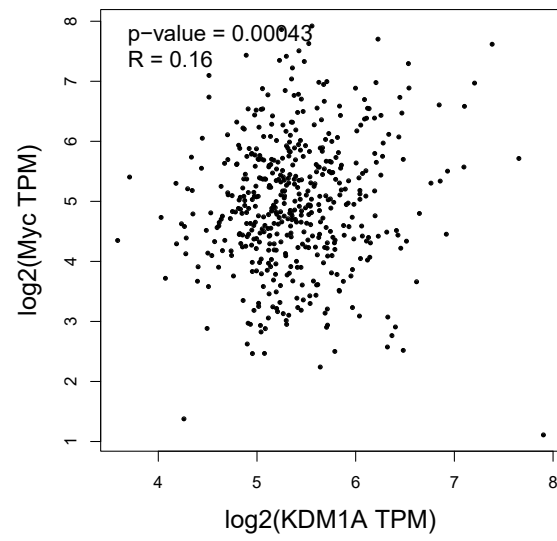

Supplement: Supplementary file 3 — Supplementary Figure S2. [file 41598_2022_23699_MOESM3_ESM.pdf]

Figure S3

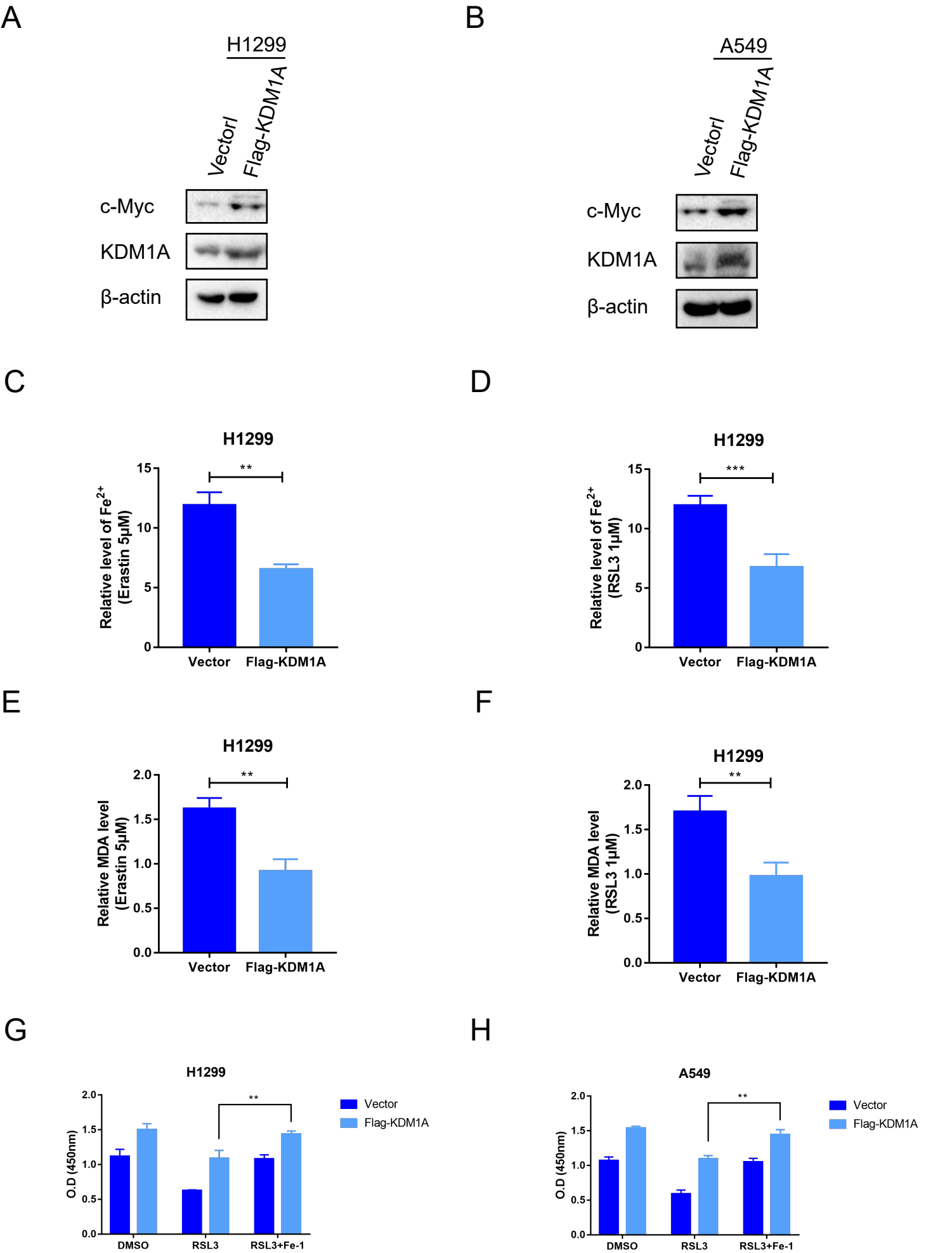

Supplement: Supplementary file 4 — Supplementary Figure S3. [file 41598_2022_23699_MOESM4_ESM.pdf]

Figure S4

A

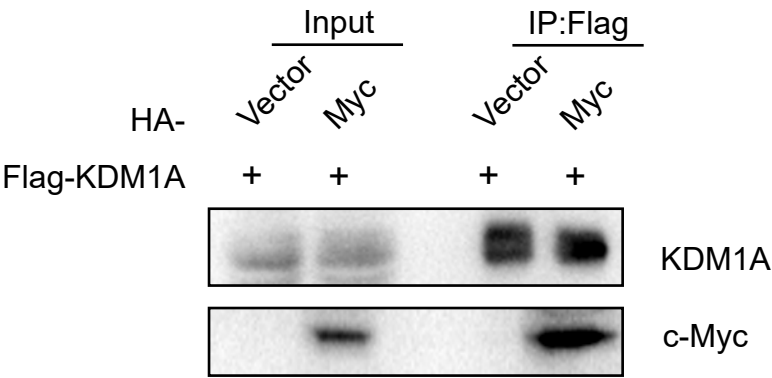

Supplement: Supplementary file 5 — Supplementary Figure S4. [file 41598_2022_23699_MOESM5_ESM.pdf]

Figure S5

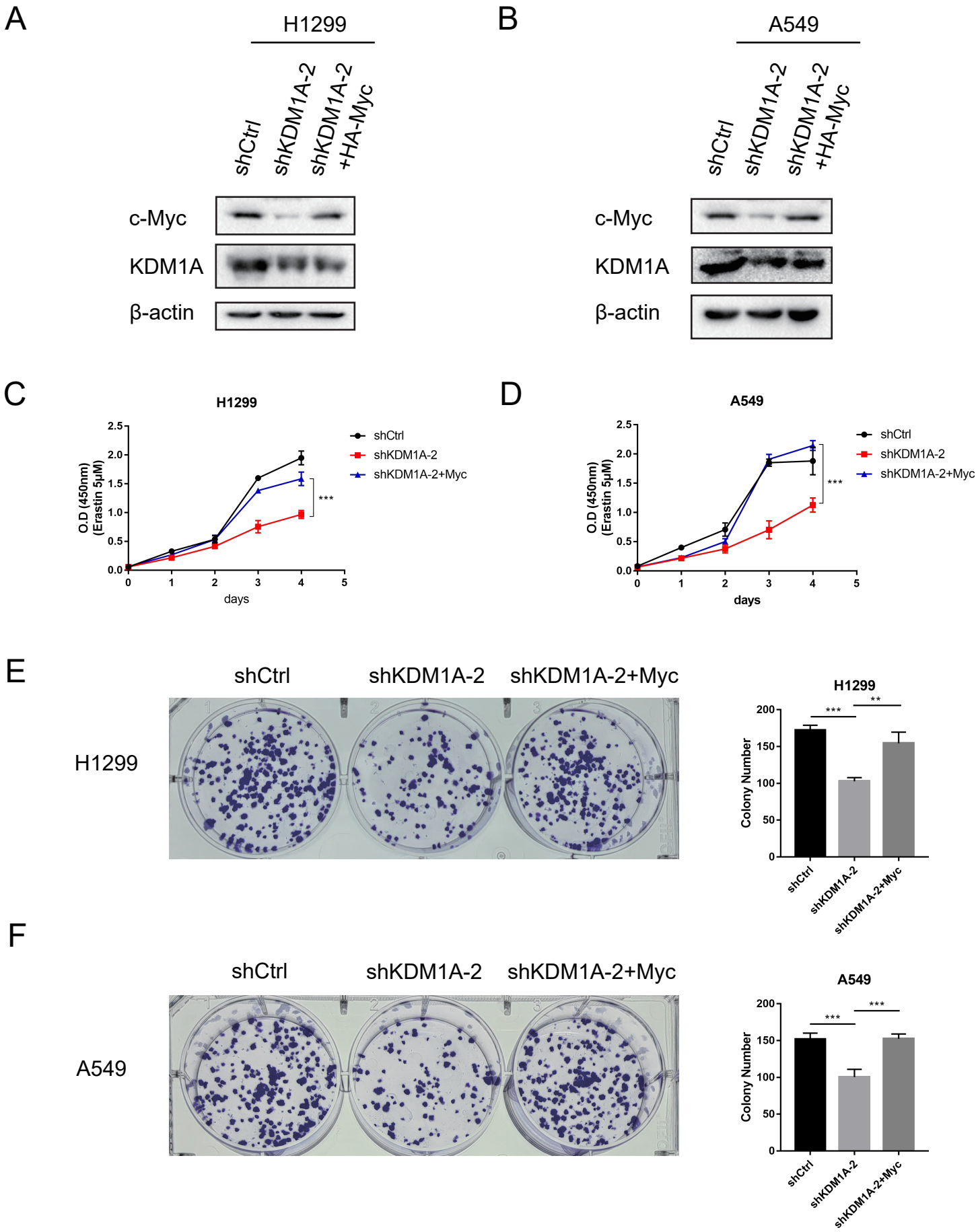

Supplement: Supplementary file 6 — Supplementary Figure S5. [file 41598_2022_23699_MOESM6_ESM.pdf]

Figure S6

A

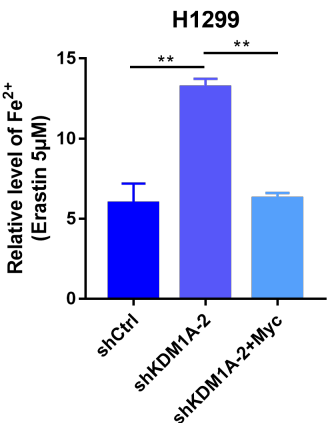

B

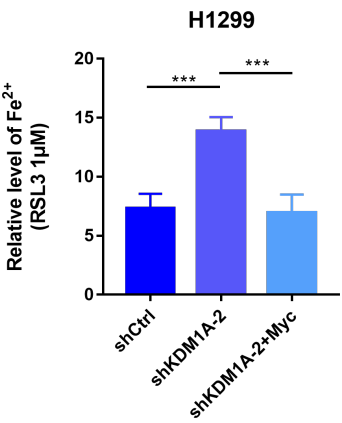

C

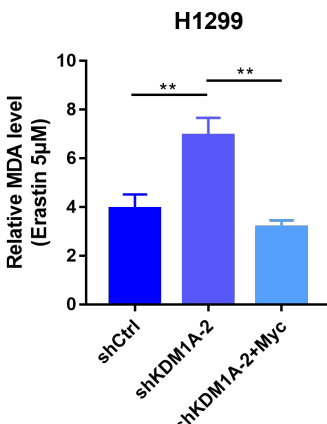

D

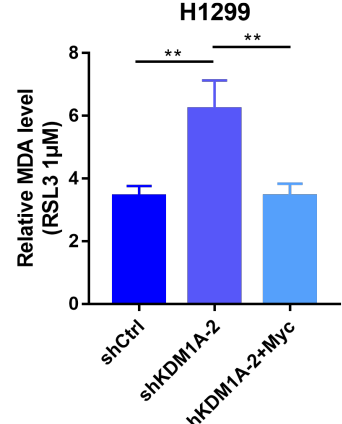

E

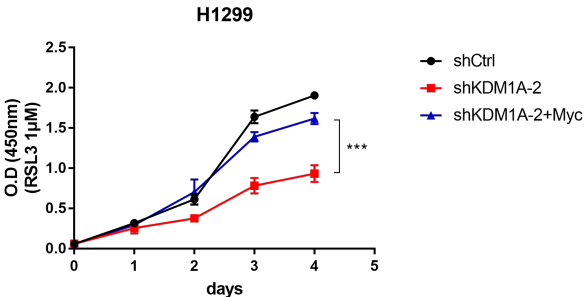

F

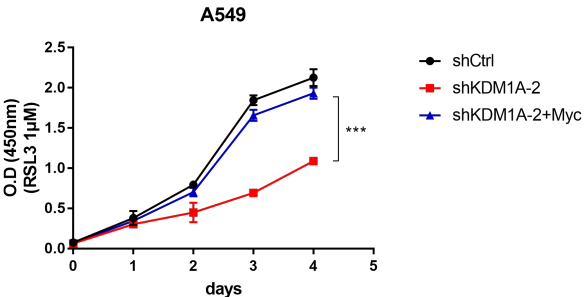

Supplement: Supplementary file 7 — Supplementary Figure S6. [file 41598_2022_23699_MOESM7_ESM.pdf]

Figure 1E

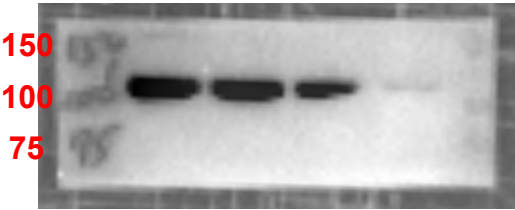

KDM1A

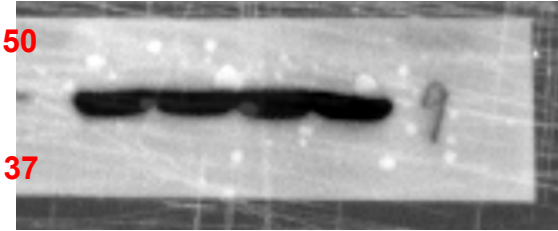

$\beta$ -actin

Supplement: Supplementary file 8 — Supplementary Figure 6. [file 41598_2022_23699_MOESM8_ESM.pdf]

Figure 3K

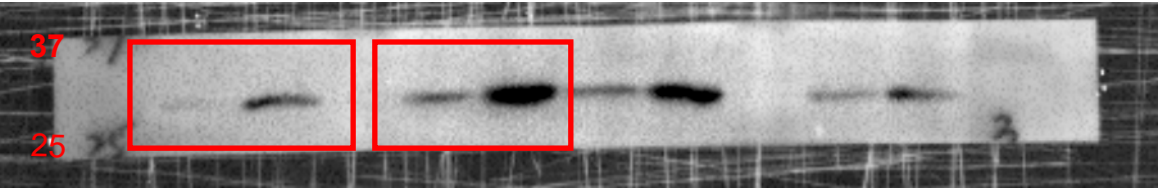

HMOX1

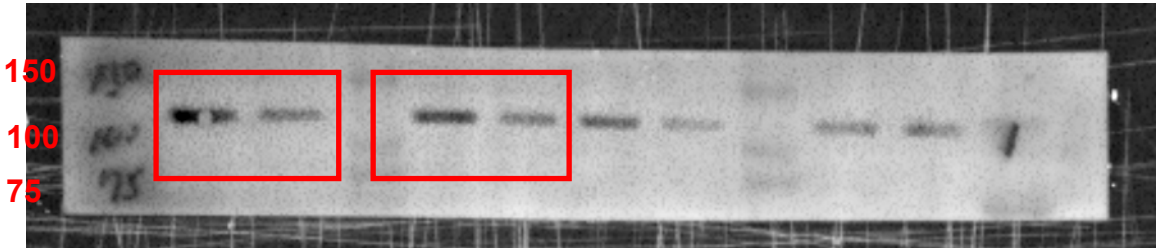

KDM1A

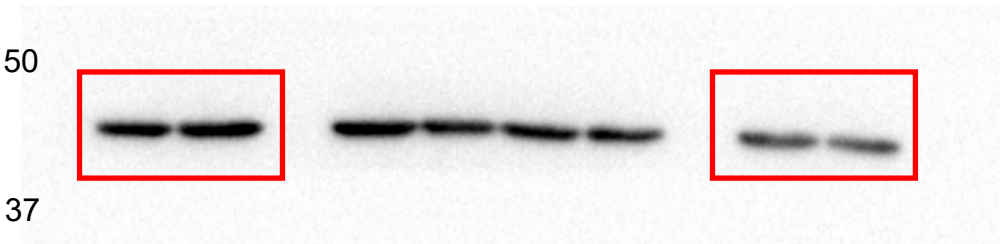

$\beta$ -actin

Supplement: Supplementary file 9 — Supplementary Figure 7. [file 41598_2022_23699_MOESM9_ESM.pdf]

Figure 4C

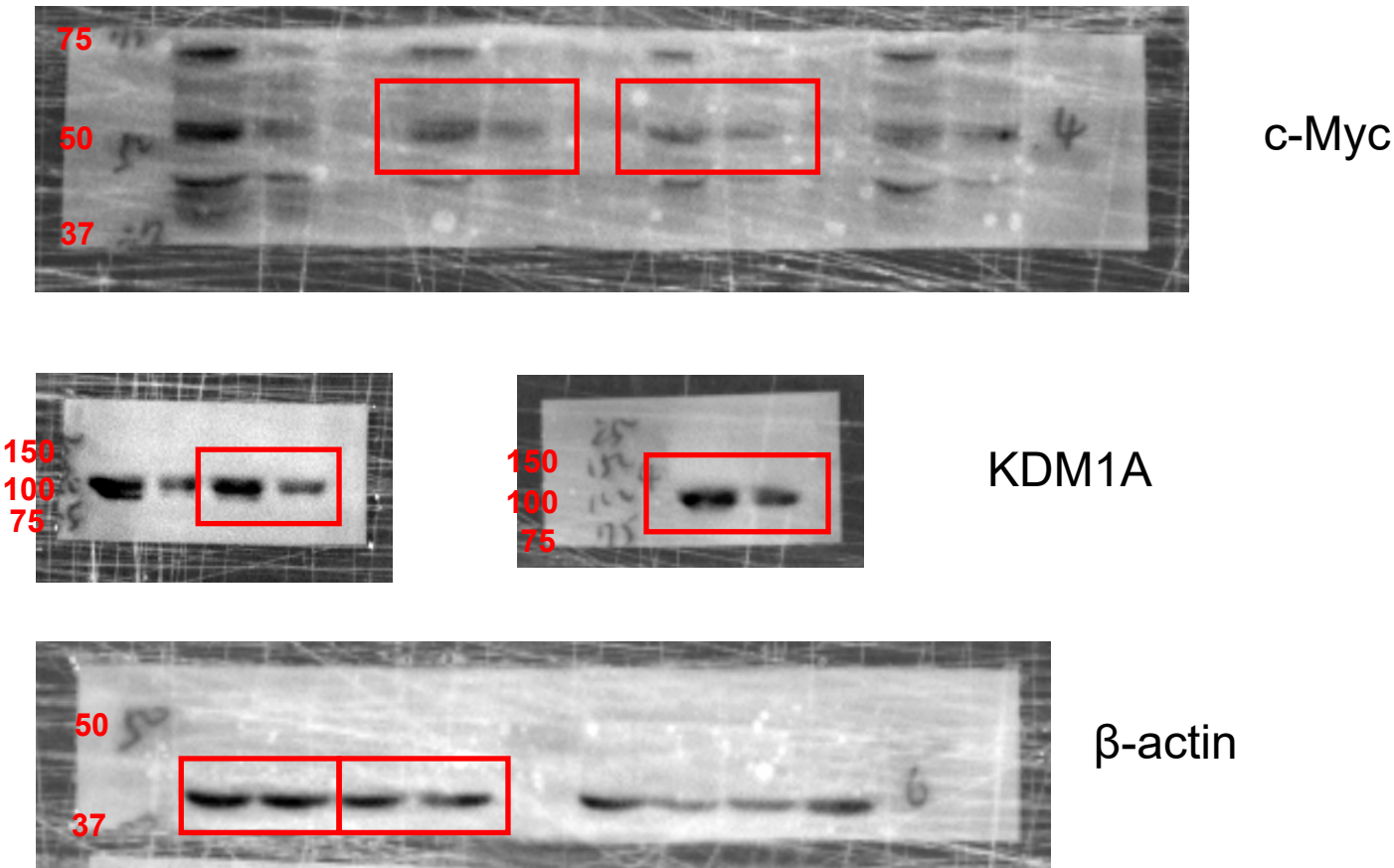

Figure 4E and 4F

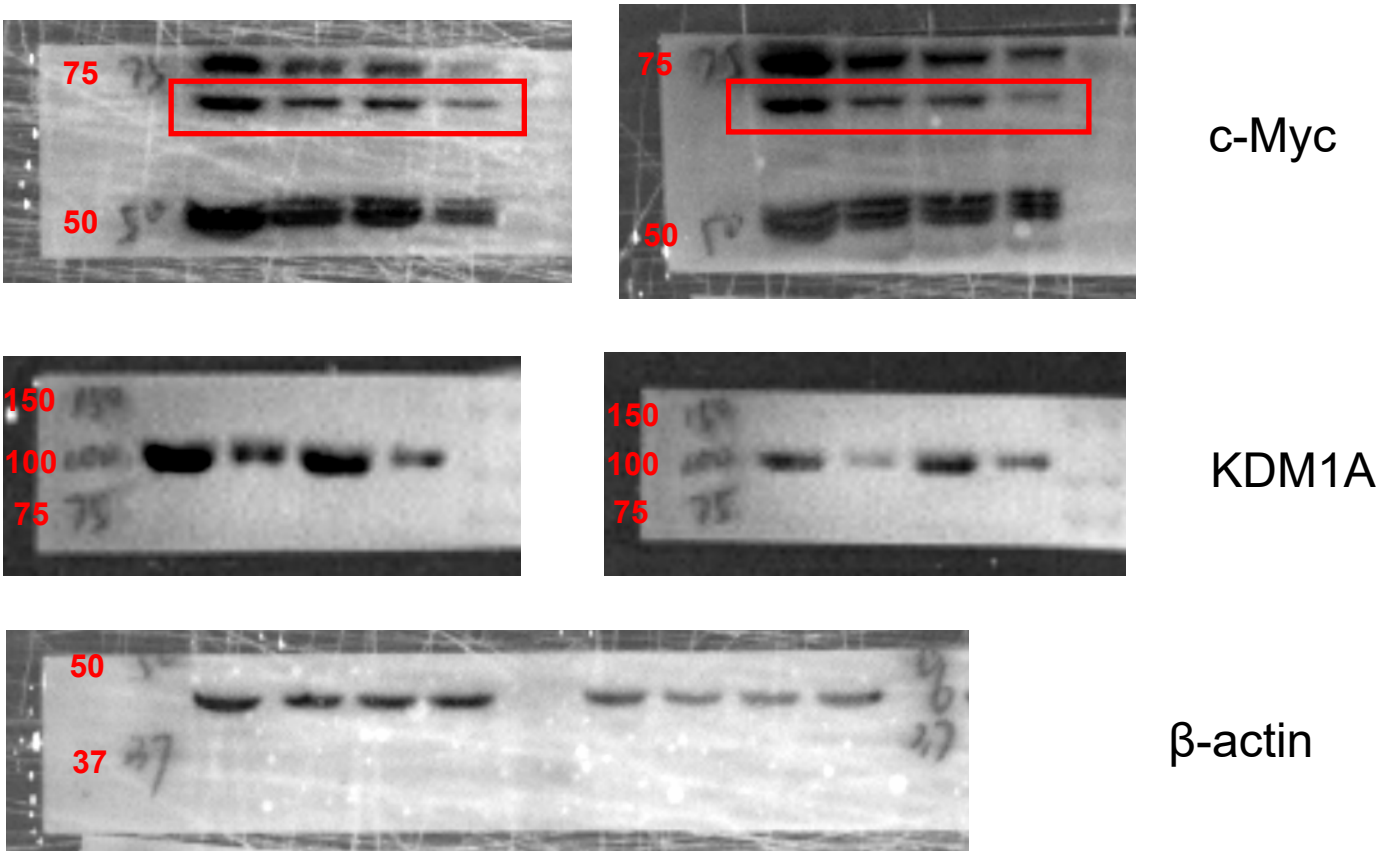

Supplement: Supplementary file 10 — Supplementary Figure 8. [file 41598_2022_23699_MOESM10_ESM.pdf]

Figure 5I and 5J

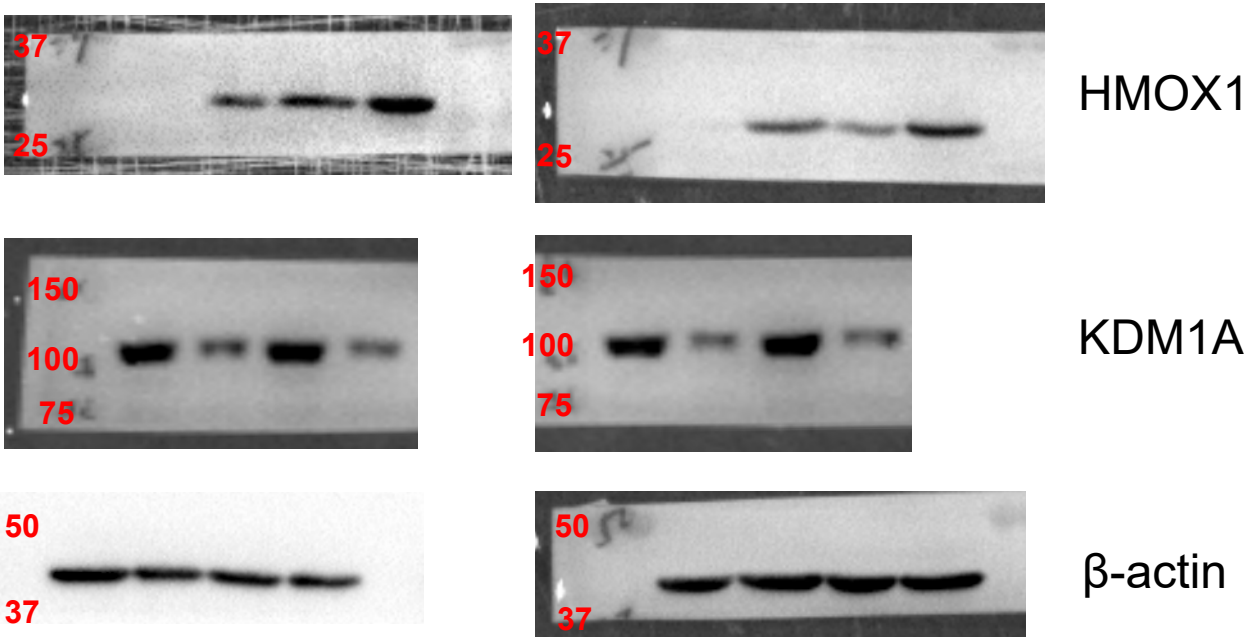

Supplement: Supplementary file 11 — Supplementary Figure 9. [file 41598_2022_23699_MOESM11_ESM.pdf]

Figure S3C-D

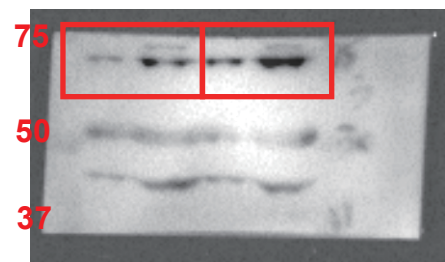

c-Myc

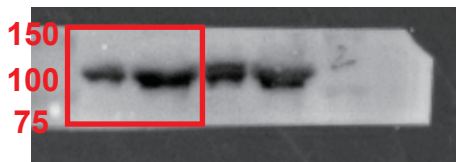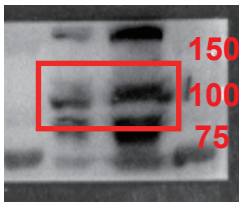

KDM1A

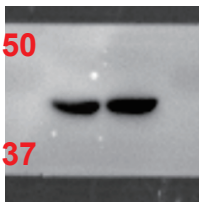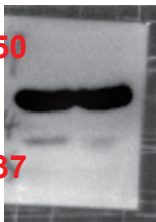

$\beta$ -actin

Supplement: Supplementary file 12 — Supplementary Figure 10. [file 41598_2022_23699_MOESM12_ESM.pdf]

Figure S4

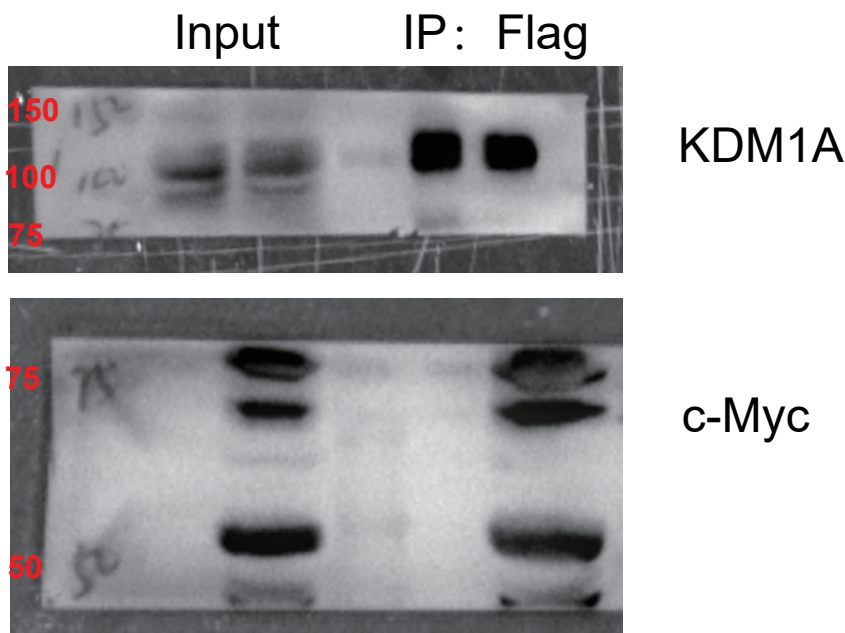

Supplement: Supplementary file 13 — Supplementary Figure 11. [file 41598_2022_23699_MOESM13_ESM.pdf]

Figure S5A-B

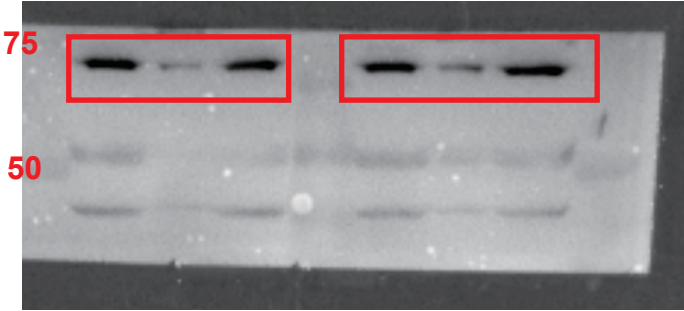

c-Myc

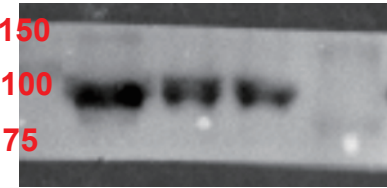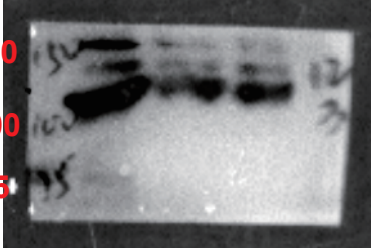

KDM1A

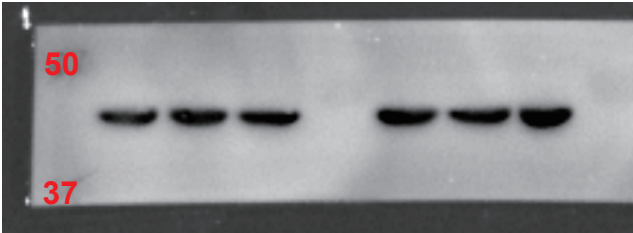

$\beta$ -actin

Supplement: Supplementary file 14 — Supplementary Figure 12. [file 41598_2022_23699_MOESM14_ESM.pdf]
