# Supplementary material for: Aberrant expression of KDM1A inhibits ferroptosis of lung cancer cells through up-regulating c-Myc
Source: Sci Rep. 2022 Nov 10;12:19168. doi: 10.1038/s41598-022-23699-4 (PMC9649633; doi:10.1038/s41598-022-23699-4)
Supplement: Supplementary file 15 — Supplementary Table S1. [file 41598_2022_23699_MOESM15_ESM.pdf]

## Figure 1A Expression of KDM1A across TCGA cancers

| | Nomal | Tumor | Statistical significance |
|---|---|---|---|
| BLCA | 19 | 408 | 1.36479999524042E-08 |
| BRCA | 114 | 1097 | <1E-12 |
| CESC | 3 | 305 | 4.719500E-02 |
| CHOL | 9 | 36 | 1.10019771071279E-11 |
| COAD | 41 | 286 | 1.62436730732907E-12 |
| ESCA | 11 | 184 | 4.39439999999625E-06 |
| GBM | 5 | 156 | 1.784400E-03 |
| HNSC | 44 | 520 | <1E-12 |
| KICH | 25 | 67 | 1.36740000000746E-06 |
| KIRC | 72 | 533 | 1.62625468647093E-12 |
| KIRP | 32 | 290 | 3.788300E-02 |
| LIHC | 50 | 371 | 1.62458935193399E-12 |
| LUAD | 59 | 515 | <1E-12 |
| LUSC | 52 | 503 | <1E-12 |
| PAAD | 4 | 178 | 8.057600E-01 |
| PRAD | 52 | 497 | 2.22860000453906E-08 |
| PCPG | 3 | 179 | 4.205200E-01 |
| READ | 10 | 166 | 4.99009999987976E-08 |
| SARC | 2 | 260 | 1.225180E-01 |
| SKCM | 1 | 472 | N/A |
| THCA | 59 | 505 | 2.748200E-01 |
| THYM | 2 | 120 | 7.796800E-01 |
| STAD | 34 | 415 | 1.62447832963153E-12 |
| UCEC | 35 | 546 | 1.62447832963153E-12 |

**Figure 1B Expression of KDM1A across CPTAC cancers**

| | Nomal | Tumor | Statistical significance |
|---|---|---|---|
| Breast cancer | 18 | 125 | 7.64989721384163E-08 |
| Colon cancer | 100 | 97 | 8.31277366348702E-31 |
| Ovarian cancer | 25 | 100 | 1.04270651949342E-10 |
| Clear cell RCC | 84 | 110 | 1.158810E-02 |
| UCEC | 31 | 100 | 2.769169E-04 |
| Lung cancer | 111 | 111 | 7.36414173529046E-16 |
| Pancreatic cancer | 74 | 137 | 4.467994E-01 |
| Head and neck cancer | 71 | 108 | 4.17511255513263E-07 |
| Glioblastoma | 10 | 99 | 7.83932312419658E-13 |
| Liver cancer | 165 | 165 | 5.27523962494378E-38 |
